# Transcriptome Analysis of the *Cf-13*-Mediated Hypersensitive Response of Tomato to *Cladosporium fulvum* Infection

**DOI:** 10.3390/ijms23094844

**Published:** 2022-04-27

**Authors:** Xiuming Jiang, Yang Li, Ran Li, Yijie Gao, Zengbing Liu, Huanhuan Yang, Jingfu Li, Jingbin Jiang, Tingting Zhao, Xiangyang Xu

**Affiliations:** Laboratory of Genetic Breeding in Tomato, Key Laboratory of Biology and Genetic Improvement of Horticultural Crops (Northeast Region), Ministry of Agriculture and Rural Affairs, College of Horticulture and Landscape Architecture, Northeast Agricultural University, Harbin 150030, China; jxm1100101@163.com (X.J.); liyang990404@163.com (Y.L.); liran3150@163.com (R.L.); gyj15603322879@163.com (Y.G.); liuzengbing2019@163.com (Z.L.); huanyaya0126@sina.com (H.Y.); lijf_2005@126.com (J.L.); jiangjingbin@neau.edu.cn (J.J.)

**Keywords:** *Solanum lycopersicum*, *Cladosporium fulvum*, RNA-seq, *Cf-13* gene, resistance response

## Abstract

Tomato leaf mold disease caused by *Cladosporium fulvum* (*C. fulvum*) is one of the most common diseases affecting greenhouse tomato production. *Cf* proteins can recognize corresponding AVR proteins produced by *C. fulvum*, and *Cf* genes are associated with leaf mold resistance. Given that there are many physiological races of *C. fulvum* and that these races rapidly mutate, resistance to common *Cf* genes (such as *Cf-2*, *Cf-4*, *Cf-5*, and *Cf-9*) has decreased. In the field, Ont7813 plants (carrying the *Cf-13* gene) show effective resistance to *C. fulvum*; thus, these plants could be used as new, disease-resistant materials. To explore the mechanism of the *Cf-13*-mediated resistance response, transcriptome sequencing was performed on three replicates each of Ont7813 (*Cf-13*) and Moneymaker (MM; carrying the *Cf-0* gene) at 0, 9, and 15 days after inoculation (dai) for a total of 18 samples. In total, 943 genes were differentially expressed, specifically in the Ont7813 response process as compared to the Moneymaker response process. Gene ontology (GO) classification of these 943 differentially expressed genes (DEGs) showed that GO terms, including “hydrogen peroxide metabolic process (GO_Process)”, “secondary active transmembrane transporter activity (GO_Function)”, and “mismatch repair complex (GO_Component)”, which were the same as 11 other GO terms, were significantly enriched. An analysis of the Kyoto Encyclopedia of Genes and Genomes (KEGG) revealed that many key regulatory genes of the *Cf-13*-mediated resistance response processes were involved in the “plant hormone signal transduction” pathway, the “plant–pathogen interaction” pathway, and the “MAPK signaling pathway–plant” pathway. Moreover, during *C. fulvum* infection, jasmonic acid (JA) and salicylic acid (SA) contents significantly increased in Ont7813 at the early stage. These results lay a vital foundation for further understanding the molecular mechanism of the *Cf-13* gene in response to *C. fulvum* infection.

## 1. Introduction

Tomato (*Solanum lycopersicum*) is one of the most widely cultivated horticultural crop species worldwide [1]. Tomato leaf mold disease caused by *Cladosporium fulvum* (*C. fulvum*) is one of the most common diseases affecting the production of tomato. *C. fulvum* is a kind of biotrophic fungus [2] of which there are many different physiological races, and this fungus mutates rapidly. *C. fulvum* infection can occur during all stages of tomato cultivation, although the fungus primarily infects seedlings, leaves, stems, and fruits, particularly under high temperature and high humidity in greenhouses and in continuously cropped fields [3]. *C. fulvum* resistance genes (*Cf* genes) can provide resistance to *C. fulvum*, and resistance genes have been identified in wild species and have been bred into the cultivated tomato. Currently, the most effective way to control tomato leaf mold disease is to use cultivars with *Cf* genes [4,5].

Throughout their evolution, plants have been affected by their environment, and various pathogenic microorganisms and have gradually developed a complete immune system. The mechanism of a plant’s innate immune system is two-tiered and involves pattern-triggered immunity (PTI) and effector-triggered immunity (ETI) [6]. PTI involves the activation of immune responses triggered by pattern recognition receptors (PRRs) on the surface of plant cells to identify conserved pathogen-associated molecular patterns (PAMPs). ETI depends on plant resistance proteins (R proteins) to identify various effectors secreted directly or indirectly by pathogenic microorganisms [7,8,9,10]. Although the downstream defense responses elicited via PTI and ETI are similar, ETI is more intense, is rapidly induced, and results in a hypersensitive response (HR) at the locally infected site. There is no clear boundary between the two processes and many studies have showed that PTI and ETI are mutually linked and, together, potentiate the immune response [11,12].

With respect to plant–pathogen interactions, different pathogens carry avirulence (*AVR*) genes that correspond to plant *R* genes and encode proteins that are recognized by effector proteins [13]. There are two types of host–pathogen interactions, a compatible system and an incompatible system, at play during tomato *C. fulvum* interactions [14]. During compatible interactions, conidia tend to germinate and form hyphae, and the hyphae grow randomly on the leaf surface. During incompatible interactions, conidia still tend to form hyphae as in compatible interactions, but plant R proteins recognize the effector proteins of pathogens, which leads to a hypersensitive response (HR). During incompatible interactions, HR could also be an outcome of elicitor recognition [15,16,17,18]. The tomato–*C. fulvum* interaction follows the typical gene-for-gene hypothesis, and the products of *C. fulvum* resistance genes (*Cf* genes) in tomato, specifically, recognize the products encoded by *AVR* genes in *C. fulvum*, activating resistance signal transduction and leading to an HR [19,20]. To date, 10 effectors have been cloned: 4 *AVR* effectors (Avr2, Avr4, Avr4E, and Avr9) in *C. fulvum* and 6 extracellular proteins (Ecp1, Ecp2, Ecp4, Ecp5, Ecp6, and Ecp7) from the infected intercellular fluid of tomato leaves [21,22,23]. At least 24 Cf genes have been reported since the 1930s [24,25], and these genes have been introduced into cultivated tomato lines [26,27,28,29,30,31,32,33,34].

With the rapid development of second-generation sequencing technology, transcriptome sequencing (RNA sequencing, RNA-seq) has contributed to groundbreaking research on gene expression, developmental regulation, and host–pathogen interactions [35,36,37,38,39,40], and has been widely applied to many different plant species such as rice [41], maize [42], and cucumbers [43], and, particularly, to tomato (carrying *Cf* genes)—*C. fulvum* interaction analyses. In preliminary studies by our group, *Cf-10*-mediated, *Cf-12*-mediated, *Cf-16*-mediated, and *Cf-19*-mediated resistance to *C. fulvum* in tomatoes has been characterized via RNA-seq [44,45,46,47]. However, few transcriptomic studies have been performed to evaluate *Cf-13*-mediated resistance. In this study, RNA-seq technology was used to analyze the *Cf-13*-mediated resistance response process, and the results lay a vital foundation for further understanding the molecular mechanism of the *Cf-13* gene in response to *C. fulvum* infection.

## 2. Results

### 2.1. Artificial Inoculation and Microscopy Analysis of C. fulvum Invasion in Ont7813 and Moneymaker (MM)

**Figure 1:**
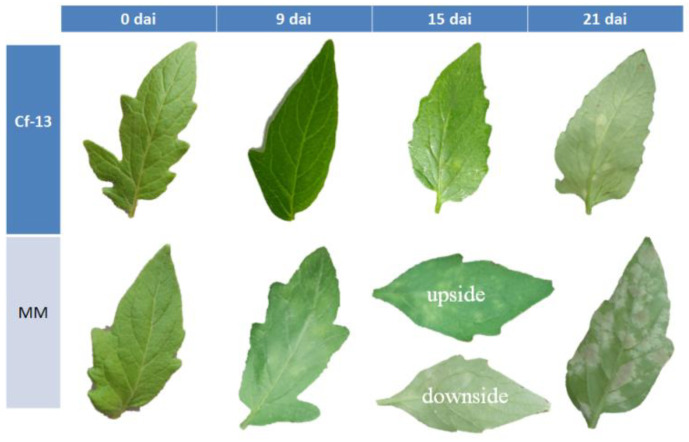
*C. fulvum* infection symptoms on the leaves of MM and *Cf-13* plants. MM: Moneymaker; *Cf-13*: Ont7813; dai: days after inoculation.

As shown in Figure 1, at 9 days after inoculation (dai), the leaves of the susceptible MM line appeared slightly chlorotic, whereas no notable signs were observed on the leaves of the resistant Ont7813 (*Cf-13*) line. At 15 dai, many substantially chlorotic spots were observed on the adaxial sides of the leaves and abundant amounts of white mold grew on the abaxial sides of the leaves of MM plants, but only a few chlorotic spots were observed on the adaxial sides of the leaves of Ont7813 plants. At 21 dai, abundant amounts of mold grew on both sides of the MM leaves, whereas only a few chlorotic spots were observed on the leaves of the Ont7813 plants. These observations showed that the artificial inoculation was successful, and based on these observations, we collected samples from each treatment at 0, 9, and 15 dai for RNA-seq and quantitative real-time PCR (qRT–PCR) analyses.

**Figure 2:**
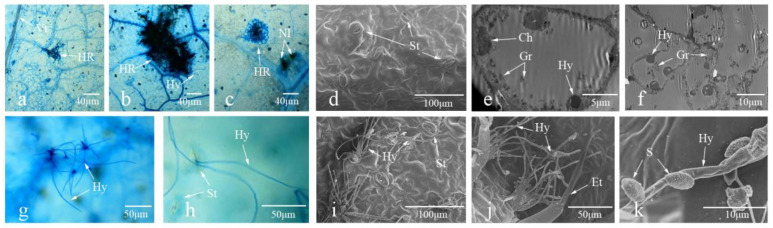
Optical microscopy, scanning electron microscopy, and transmission electron microscopy images of tomato leaf samples. (**a**–**c**): Ont7813 leaf stained with lactophenol trypan blue at 9, 15, and 21 dai, as seen under an optical microscope. (**d**–**f**): Ont7813 leaf at 9, 15, and 21 dai under an electron microscope. (**g**,**h**): MM leaves stained with lactophenol trypan blue at 9 and 15 dai, as seen under an optical microscope. (**i**–**k**): MM leaf at 9, 15, and 21 dai under an electron microscope. Hy: hypha; Nl: necrotic lesion; S: spore; St: stoma; Et: epidermal trichome; Ch: chloroplast; Gr: granum.

The infection processes of *C. fulvum* in the leaves of Ont7813 and MM were observed via optical microscopy, scanning electron microscopy, and transmission electron microscopy (Figure 2). As shown in Figure 2, in Ont7813 leaves, some small HR areas were visible at 9 dai (Figure 2a), and the HR areas seemingly expanded at 15 dai (Figure 2b). At this point, many substantial HR areas were observed, and these areas of the leaves formed necrotic lesions at 21 dai (Figure 2c). There was no conidial germination at 9 dai, as seen under a scanning electron microscope (Figure 2d). Under a transmission electron microscope, a small number of hyphae appeared to destroy chloroplast membranes and chloroplasts at 15 dai (Figure 2e), and a great number of hyphae appeared to destroy a large number of chloroplast membranes at 21 dai (Figure 2f), which limited the further growth of hyphae. In MM leaves, hyphae germinated (Figure 2g) and emerged through the stomata at 9 dai (Figure 2h), and the growth and number of emergent hyphae continued to increase from 9 to 21 dai, as seen under a scanning electron microscope (Figure 2i–k), which resulted in unrestricted fungal growth in the MM leaves.

### 2.2. Illumina Sequencing, Mapping Reads, and Transcript Identification

In this study, 18 samples were sequenced using an Illumina HiSeq platform, with an average of 3.87 Gb of clean data generated. As shown in Table 1, more than 97% of clean reads were >Q20, and 92% of clean reads were >Q30. After filtering, 22.20–30.77 million clean reads were generated, and at least 93.73% of these reads were mapped to the tomato reference genome (ITAG 4.0), among which more than 89.90% were aligned to unique mapped reads. After using StringTie to reconstruct the transcript, we identified 696 novel genes that were not annotated in the reference genome (or set of reference genes). Pearson correlation coefficients were calculated for all gene expression values between each pair of samples, and the correlation coefficients of 18 datasets (34,772 genes in total) are presented as heatmaps (Figure S1).

### 2.3. Identification of Differentially Expressed Genes (DEGs)

**Figure 3:**
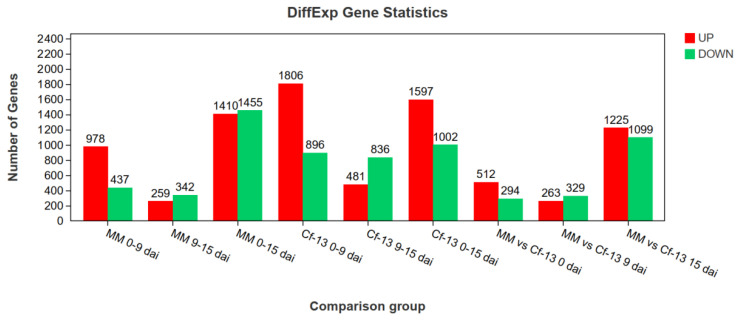
The DEGs among different comparison groups. 0–9 dai: comparison between 0 and 9 dai; 9–15 dai: comparison between 9 and 15 dai; 0–15 dai: comparison between 0 and 15 dai.; dai: days after inoculation.

Using DESeq2 software [48], genes were considered differentially expressed (Figure 3) between samples when the false discovery rate (FDR) was < 0.05 and the |log2(fold change [FC])| was >1. Between 0 and 9 dai with *C. fulvum*, there were significantly more DEGs in the Ont7813 plants than in the MM plants, and there were twice as many upregulated DEGs as downregulated DEGs in both the MM and Ont7813 plants. Between 9 and 15 dai with *C. fulvum*, the number of upregulated DEGs decreased significantly, and the number of downregulated DEGs changed little in both the MM and Ont7813 plants. During the entire infection with *C. fulvum*, the number of downregulated DEGs was slightly higher than that of unregulated DEGs in the MM plants, whereas the number of upregulated DEGs was much greater than that of downregulated DEGs in the Ont7813 plants. When the Ont7813 plants were compared with the MM plants, these DEGs were decreased from 0 dai to 9 dai, and then increased significantly from 9 dai to 15 dai.

**Figure 4:**
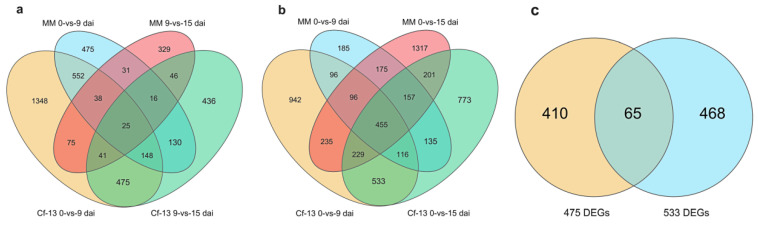
Venn diagrams of the different groups of DEGs. (**a**) Venn diagram of DEGs among the MM 0 vs. 9 dai, MM 9 vs. 15 dai, *Cf-13* 0 vs. 9 dai, and *Cf-13* 9 vs. 15 dai comparisons. (**b**) Venn diagram of DEGs among the MM 0 vs. 9 dai, MM 0 vs. 15 dai, *Cf-13* 0 vs. 9 dai, and *Cf-13* 0 vs. 15 dai comparisons. (**c**) Venn diagram of the 475 DEGs and 533 DEGs comparison (MM 0 vs. 9 dai, MM 9 vs. 15 dai, MM 0 vs. 15 dai, *Cf-13* 0 vs. 9 dai, *Cf-13* 9 vs. 15 dai, and *Cf-13* 0 vs. 15 dai). *Cf-13* and MM tomatoes were inoculated with *C. fulvum* and sampled at 0, 9, and 15 dai. 475 DEGs: the common genes differentially expressed between 0–9 dai and 9–15 dai only in *Cf-13*; 533 DEGs: the common genes differentially expressed between 0–9 dai and 0–15 dai only in *Cf-13*.

To more intuitively analyze the relationship of DEGs between different comparisons, we constructed Venn diagrams of the genes that were differentially expressed between the different comparisons (Figure 4). Numerous common DEGs were detected between Ont7813 and MM, which indicates that some DEGs in the comparison group are not involved in the leaf mold disease resistance process (Figure 4a,b). Furthermore, 475 common DEGs were unique to Ont7813 at *Cf-13* 0 vs. 9 dai and *Cf-13* 9 vs. 15 dai (Figure 4a), whereas 533 common DEGs were unique to Ont7813 at *Cf-13* 0 vs. 9 dai and *Cf-13* 0 vs. 15 dai (Figure 4b). These DEGs are likely to participate in the leaf mold disease resistance process. According to a Venn diagram comparing the 475 DEGs and 533 DEGs (Figure 4c), 943 DEGs were unique to Ont7813 disease-resistant materials, and these genes not only participate in the early response, but also play a continuous role in subsequent regulatory processes.

### 2.4. Validation of DEG Expression Patterns

**Figure 5:**
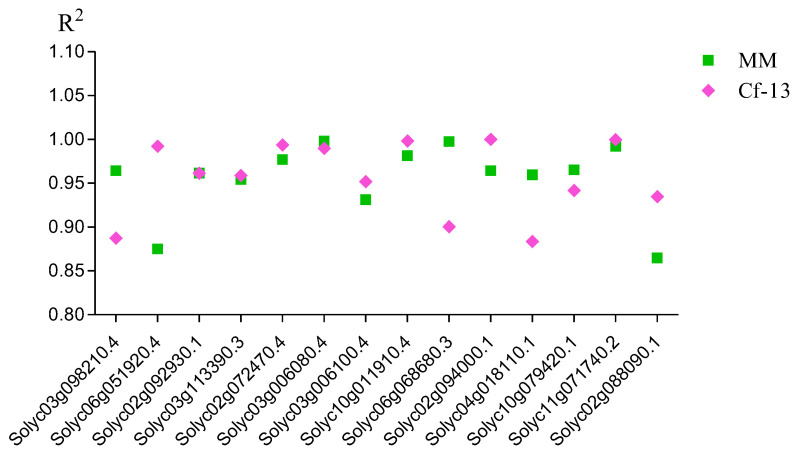
Correlation coefficients between the RNA-seq and qRT–PCR results. The expression patterns for both *Cf-13* (0, 9, and 15 dai) and MM (0, 9, and 15 dai) were analyzed for each gene. The results obtained from both methods (RNA-seq and qRT–PCR analysis) were used to calculate correlation coefficients (R^2^ values). MM: Moneymaker; *Cf-13*: Ont7813.

To verify the RNA-seq data, quantitative real-time PCR (qRT–PCR) was used to analyze the expression patterns of 14 DEGs that were selected from the significantly enriched Kyoto Encyclopedia of Genes and Genomes (KEGG) pathways (such as “plant–pathogen interaction” and “MAPK signaling pathway-plant”). Correlation coefficients between the RNA-seq and qRT–PCR results were calculated, and these genes tended to show a significant positive correlation between the RT–qPCR results and RNA-seq data (the R^2^ values were between 0.86 and 1.0), indicating that the RNA-seq data were reliable (Figure 5).

### 2.5. Gene Ontology (GO) Enrichment and KEGG Pathway Analysis of DEGs

**Figure 6:**
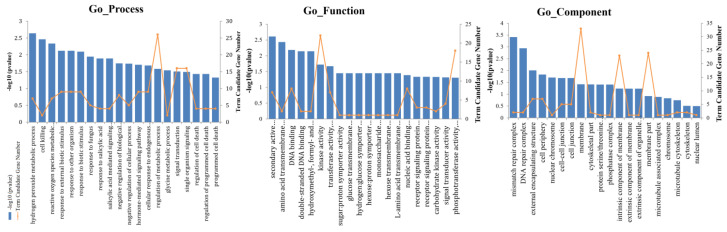
GO enrichment of 943 genes differentially expressed specifically in the Ont7813 response process.

The GO system [49] is an international standardized gene functional classification system in which a dynamically updated, controlled vocabulary and a strictly defined concept are used to comprehensively describe properties of genes and their products in any organism. To determine the functions of DEGs involved in the response to *C. fulvum* between MM and Ont7813, we analyzed assigned GO terms with GO-seq [50]. The GO classification results are shown in Figure S2. DEGs were enriched in three ontologies: biological processes (BPs), molecular functions (MFs), and cellular components (CCs). Compared with Ont7813, MM presented more genes associated with almost all GO terms, except for the GO term “cell killing” in the BP category in response to *C. fulvum*. For the 943 differentially expressed genes specifically in Ont7813 (Figure 6), significant GO terms were enriched in “hydrogen peroxide metabolic process”, “cell killing”, “reactive oxygen species metabolic process”, “response to external biotic stimulus”, “response to other organism”, and “response to biotic stimulus” in the BP category, and these terms are associated with disease resistance. In the MF category, the top three enriched terms were “secondary active transmembrane transporter activity”, “amino acid transmembrane transporter activity”, and “DNA binding”, and in the CC category, the significantly enriched terms included “mismatch repair complex”, “DNA repair complex”, and “external encapsulating structure”.

**Figure 7:**
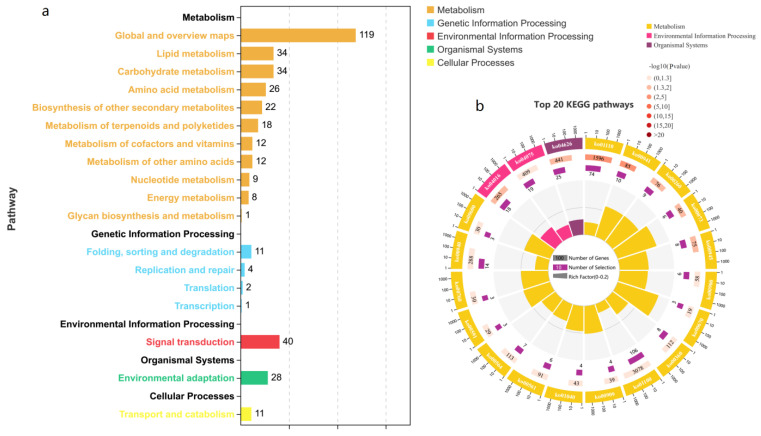
KEGG pathway enrichment of 943 genes differentially expressed specifically during the Ont7813 response process. (**a**) Statistics of the DEGs in different pathways. (**b**) Enrichment of the top 20 KEGG pathways.

Genes typically interact to play roles in certain biological functions. A pathway-based analysis can help to further understand the biological functions of genes. The KEGG pathway enrichment analysis revealed metabolic pathways and signal transduction pathways significantly enriched in DEGs compared with the whole-genome background. Pathways with *p* values of <0.05 were screened and used for comparative analyses. The top 20 KEGG pathway IDs and names are shown in Table S1. As shown in Figure 7a, the 943 DEGs were enriched in 5 KEGG_A_Class pathways, including “metabolism”, “genetic information processing”, “environmental information processing”, “organismal systems”, and “cellular processes”. “Metabolism” had the most DEGs, followed by “environmental information processing” and “organismal systems”. As shown in Figure 7b, the top 20 pathways included 3 KEGG_A_Class pathways: “metabolism”, “environmental information processing”, and “organismal systems”. In metabolism, the following pathways were significantly enriched: “biosynthesis of secondary metabolites”, “flavonoid biosynthesis”, “glycine, serine and threonine metabolism”, and 14 others. In environmental information processing, the pathways “MAPK signaling pathway–plant” and “plant hormone signal transduction” were significantly enriched. In the organismal systems, the pathway “plant–pathogen interaction” was significantly enriched; in addition, compared with those pathways, “biosynthesis of secondary metabolites” and “metabolic pathways” were enriched in more DEGs and were related to the Ont7813 tomato response to *C. fulvum* infection. In summary, these pathways may be the major metabolic pathways involved in the *Cf-13*-mediated resistance response to *C. fulvum*.

### 2.6. Identification of Key Regulatory Genes Involved in Important Pathways

**Figure 8:**
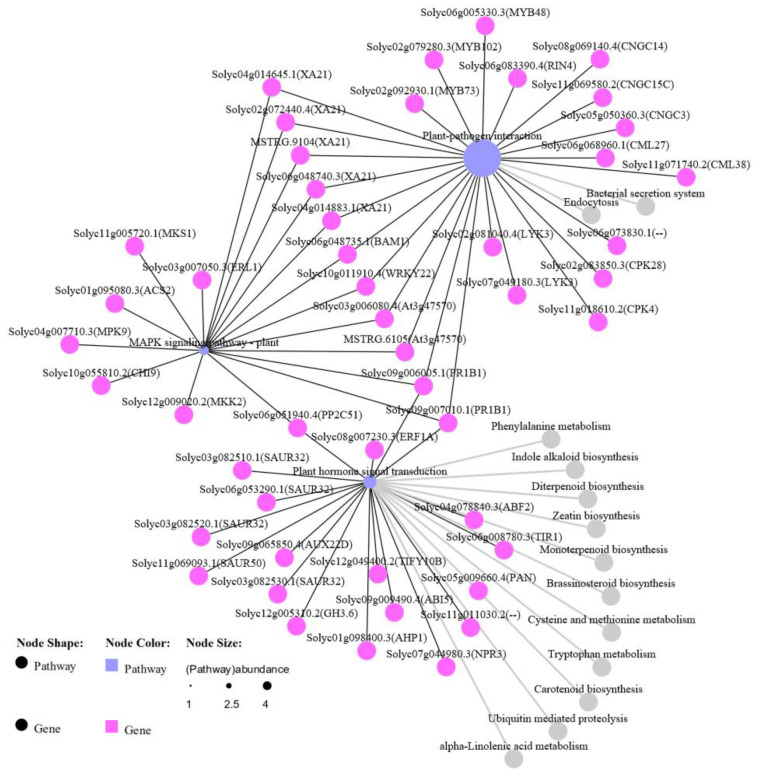
Network diagram of key KEGG pathways. The KEGG pathways included the “plant–pathogen interaction”, “MAPK signaling pathway–plant”, and “plant hormone signal transduction” pathways, and the gray pathways are the relevant ones.

The “plant–pathogen interaction”, “MAPK signaling pathway–plant”, and “plant hormone signal transduction” pathways were identified as being significantly enriched in the Ont7813 plants. Moreover, because these pathways are vital pathways in plant–pathogen interaction studies, we focused on these three pathways and constructed a network diagram (Figure 8). The network of KEGG pathways showed that the pathways were strongly related. Twenty-five, eighteen, and nineteen DEGs were identified in the “plant–pathogen interaction”, “MAPK signaling pathway–plant”, and “plant hormone signal transduction” pathways, respectively. Eleven DEGs were shared between the “plant–pathogen interaction” pathway and the “MAPK signaling pathway–plant” pathway, three DEGs were shared between the “MAPK signaling pathway–plant” pathway and the “plant hormone signal transduction” pathway, and two DEGs were shared between the “plant–pathogen interaction” pathway and the “plant hormone signal transduction” pathway. We also found two unique genes in all three KEGG pathways, the *PR-1* gene (*Solyc09g006005.1* and *Solyc09g007010.1*), which is a well-known resistance-related gene in plants. The common genes in these three KEGG pathways further revealed that different pathways ultimately produced a combined effect in the *Cf-13*-mediated resistance response to *C. fulvum*.

**Figure 9:**
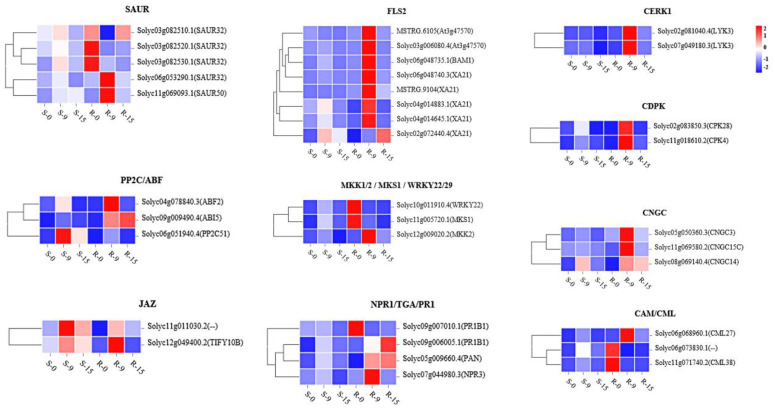
Expression patterns of genes a certain number of days after inoculation.

Further analysis of the key regulatory genes showed enrichment in important pathways, and the details of the comparison process and information are indicated in the pathway maps from the KEGG analysis (map04626, plant–pathogen interaction; map04016, MAPK signaling pathway–plant; and map04075, plant hormone signal transduction). As shown in Figures S3–S5, “CERK1”, “CDPK”, “CNGC”, and “CAM/CML” were identified in the “plant–pathogen interaction” pathway, and “FLS2”, “WRKY22/29”, “MKK1/2”, and “MKS1” were identified in the “MAPK signaling pathway–plant” pathway. Similarly, in the “plant hormone signal transduction” pathway, “SAUR” was identified in the “tryptophan metabolism” pathway, “PP2C” and “ABF” were identified in the “carotenoid biosynthesis” pathway, “JAZ”, was identified in the “α-linolenic acid metabolism” pathway, and “NPR1”, “TGA”, and “PR1” were identified in the “phenylalanine metabolism” pathway. To further analyze the expression patterns of these genes in resistant and susceptible plants, detailed expression information was collected, which is shown in Figure 9 as heatmaps (Tables S3–S5). The key regulatory genes exhibited different expression patterns between resistant plants and susceptible plants. Most of the key genes demonstrated significantly upregulated expression from 0 to 9 dai and downregulated expression from 9 to 15 dai, but the expression of a small portion of the genes steadily rose from 0 to 15 dai, particularly in the resistant plants. In the susceptible plants, most regulatory genes were downregulated, such as “FLS2” and “MKK1/2, MKS and WRKY22/29” in the “MAPK signaling pathway–plant” pathway, “NPR1, TGA and PR1” in the “plant hormone signal transduction” pathway, and almost all the genes in the “plant pathogen interaction” pathway.

### 2.7. Measurements of Jasmonic Acid (JA) and Salicylic Acid (SA)

**Figure 10:**
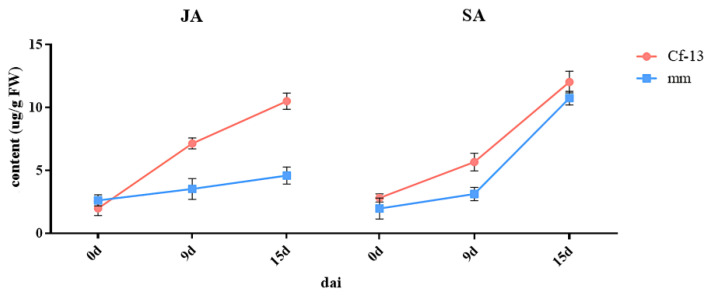
Fluctuations in JA and SA as a function of days after inoculation in *Cf-13* plants and MM plants. JA: jasmonic acid; SA: salicylic acid; *Cf-13*: Ont7813; MM: Moneymaker; dai: days after inoculation.

JA and SA are very important hormones involved in plant immunity. To explore the hormone response to *C. fulvum* infection, high-performance liquid chromatography tandem mass spectrometry (HPLC–MS/MS) was used to measure the SA and JA contents. The results showed that the SA and JA contents fluctuated in the different plants in response to inoculation (Figure 10). The JA content dramatically differed between the resistant and susceptible plants. The JA content continuously increased to a very high level from 0 to 15 dai, and the rate of increase from 0 to 9 dai was greater than that from 9 to 15 dai in Ont7813, whereas it was relatively stable from 0 to 15 dai in MM. The SA content increased slightly from 0 to 9 dai and then rapidly increased, peaking from 9 to 15 dai, in both Ont7813 and MM. Although the change in SA content was similar between the resistant and susceptible plants, the SA content of the resistant plants was slightly higher than that of the susceptible plants.

## 3. Discussion

In this study, we first observed the phenotypes of leaves from Ont7813 and MM after inoculation, and based on a modified Faoro’s method [51], we used lactophenol trypan blue staining to document the interaction process of *C. fulvum* with resistant plants and susceptible plants through microscopic observations. In Ont7813 leaves, some small HR areas were visible at 9 dai (Figure 2), whereas in MM leaves, hyphae emerged and continued to increase. These findings were consistent with our group’s previous studies of other *Cf* genes [44,45,46,47]. During the entire process of plant–pathogen interactions, 9 dai is an important time point, and the entire process can be separated into two stages: an early stage and a late stage. Based on the different stages and different materials, an analysis between and within the two groups was performed. Between the two groups, there were significantly more DEGs in the Ont7813 plants than in the MM plants, and there were more upregulated DEGs than downregulated DEGs in both the MM and Ont7813 plants at the early stage. However, fewer DEGs were detected in the MM plants than in the Ont7813 plants, and there were more downregulated DEGs than upregulated DEGs in both the MM and Ont7813 plants at the late stage. Within the two groups, in both the MM plants and the Ont7813 plants, more DEGs were detected at the early stage than at the late stage, the expression of the upregulated DEGs was substantially decreased, and the downregulated DEGs showed a slight decline from the early stage to the late stage. In summary, gene expression changed most actively at the early stage of plant–pathogen interactions, and similar results have been reported for other *Cf* genes. DEGs in the MM vs Ont7813 group were decreased at the early stage, and then increased significantly at the late stage. The trend of DEGs in the MM vs Ont7813 group showed that both compatible and incompatible systems made a response to the pathogen infection at the early stage, and so the number of DEGs decreased. As the infection continued, more downstream defense response were triggered in the incompatible system and the number of DEGs significantly increased.

To more intuitively analyze the relationships of DEGs, Venn diagrams of the DEGs between different comparisons were constructed (Figure 4). A total of 943 DEGs were identified specifically in the Ont7813 response process. Through GO classification analysis, 14 GO terms were significantly enriched, including “cell killing”, which was specifically enriched in the BP category during the response to *C. fulvum*, and most Go terms were associated with disease resistance. In the *Cf-19*-mediated resistance response process, the GO term “cell killing” was also specifically enriched in the resistant line [47]. Furthermore, the KEGG enrichment analysis indicated that DEGs were significantly enriched in “plant–pathogen interaction”, “MAPK signaling pathway–plant”, “plant hormone signal transduction”, and “metabolic pathways”. Other studies have shown that in *Cf-16*-mediated resistance, the response process indicated that most of the DEGs were classified into “Plant hormone signal transduction” and “Plant-pathogen interaction” [46], while during the *Cf-12*-mediated resistance response process, DEGs were significantly enriched in defence-signalling pathways such as the calcium-dependent protein kinase pathway and the jasmonic acid signalling pathway [45]. In the *Cf-10*-mediated resistance response process, the majority of DEGs were associated with defense-signaling pathways, including oxidation–reduction processes, oxidoreductase activity, and plant hormone signal transduction [44]. In summary, GO and KEGG enrichment analyses demonstrated that most DEGs were enriched in disease resistance-related GO terms and pathways, and future studies should focus on the detailed comparisons of *Cf-10*-, *Cf-12*-, *Cf-16*-, *Cf-1-9*, and *Cf-13*-mediated resistance responses to *C. fulvum* invasion.

Plant hormones are well known as important regulatory signals of defense responses in plants. JA and SA are hormones that participate in plant defense responses. In our study, we measured JA and SA contents at 0, 9, and 15 dai (Figure 10). The JA contents continuously increased to a very high level from 0 to 15 dai, increasing more rapidly in the early stage than in the late stage in the resistant plants. The SA contents increased slightly at the early stage, and then rapidly increased to their highest levels at the late stage in both Ont7813 and MM, but the SA contents of the resistant plants were always higher than those of the susceptible plants. Nineteen DEGs were identified in the significantly enriched KEGG pathway “plant hormone signal transduction” (Table S5). Furthermore, the gene expression patterns of the “JAZ” genes (*Solyc11g011030.2* and *Solyc12g049400.2*) were upregulated significantly in the resistant plants at the early stage, and “JAZ” also found to be upregulated in *Cf-12*, *Cf-16*, and *Cf-19* tomato plants after *C. fulvum* infection [44,45,47]. “JAZ” is the key regulatory point we screened in this study. The gene expression patterns of NPR1 (*Solyc07g044980.3*), TGA (*Solyc05g009660.4*), and PR-1 (*Solyc09g006005.1* and *Solyc09g007010.1*) were involved in the SA signaling pathway. NPR1 is a node through which the SA and JA pathways intersect [52]. Biotrophs or hemibiotrophs can live within their host tissue without causing immediate death, as has been shown for *Pseudomonas syringae* in the intracellular space [53]. *C. fulvum* is a type of biotrophic fungus. In general, insects and necrotrophs trigger jasmonate production, and biotrophs trigger salicylate production. JAZs are direct targets of the SCF^COI1^ E3 ubiquitin ligase and negatively regulate the key transcriptional activator of jasmonate responses, MYC2 [52]. There is extensive crosstalk between the JA and SA signaling pathways, and JAZ protein expression suppresses JA activation and indirectly promotes SA activation. The genes involved in the JA and SA signaling pathways may have important functions in the response of *Cf-13* to *C. fulvum* infection. Aside from JA and SA, DEGs related to four other hormones, i.e., auxin, cytokinin, abscisic acid, and ethylene, were detected, which reveals a complex signal transduction network. The SAUR gene involved in the “tryptophan metabolism” pathway and the PP2C and ABF genes involved in the “carotenoid biosynthesis” pathway were involved in plant cell enlargement and cell division. In summary, the cooperation of all these key genes formed a resistance process between *Cf-13* and *C. fulvum*.

The Ca^2+^ signaling process is known to regulate plant immunity. Constitutive cytoplasmic Ca^2+^ influx induces constitutive defense activation and cell death [54]. In our study, the expression patterns of the CaM/CML genes were different between resistant plants and susceptible plants, and these genes were upregulated in the susceptible plants but not in the resistant plants (Table S3). These changes suggest that Ca^2+^ signal transduction differs between resistant plants and susceptible plants at the early stage of pathogen infection. Throughout their evolution, plants have gradually developed a complete immune system [6]. The first layer of induced immunity, PTI, is activated by the recognition of pathogen-associated molecular patterns (PAMPs). The PTI signaling defense response, which includes activation of MAPK kinase cascades, cytoplasmic Ca^2+^ influx, production of reactive oxygen species (ROS), and expression of defense genes, is then activated. However, pathogens deploy effectors to suppress PTI. When they are recognized by NB-LRRs known as NLRs, the second immune layer, ETI, is activated. NLRs directly or indirectly perceive pathogenic effectors, leading to a series of intracellular signaling events such as the production of the stress hormone salicylic acid (SA), the production of reactive oxygen species (ROS), the hypersensitive response (HR), the expression of pathogenesis-related (PR) proteins and systemic signals, and systemic acquired resistance (SAR) [55]. The Cf/Avr interaction is a model for ETI study; however, in our research, the expression levels of CERK1 (*Solyc02g081040.4* and *Solyc07g049180.3*) DEGs, CDPK (*Solyc02g083850.3* and *Solyc11g018610.2*) DEGs, and CNGC (*Solyc05g050360.3*, *Solyc08g069140.4* and *Solyc11g069580.2*) DEGs, which encode important components of PTI, were upregulated at the early stage in the resistant plants. FLS2 (*MSTRG.6105*, *MSTRG.9104*, *Solyc02g072440.4*, *Solyc03g006080.4*, *Solyc04g014645.1*, *Solyc04g014883.1*, *Solyc06g048735.1*, and *Solyc06g048740.3*) genes were detected in the “plant–pathogen interaction” and “MAPK signaling pathway–plant” pathways, and the expression patterns of FLS2 genes were markedly upregulated at the early stage in the resistant plants (Table S4). With FLS2 recognizing flg22, downstream signaling pathways such as the defense response of cell death, camalexin synthesis, and the defense response of pathogens (PR1, *Solyc09g006005.1* and *Solyc09g007010.1*) were induced. Finally, our results illustrate that PTI and ETI are mutually linked and, together, potentiate the immune response between *Cf-13* plants and *C. fulvum*.

## 4. Materials and Methods

### 4.1. Plant Materials and C. fulvum Infection

The resistant line Ont7813 (carrying the *Cf-13* gene) and the susceptible line MM (carrying the *Cf-0* gene) were kindly provided by staff at the Chinese Academy of Agricultural Sciences. The genetic background of Ont7813 was a related species wild accession of tomato. The two tomato cultivars were grown at the Horticultural Experimental Station of Northeast Agricultural University (Harbin, China). At the 4–6 leaf stage, thirty seedlings per line were sprayed with a conidial suspension of *C. fulvum* race 1.2.3.4 at 1 × 10^7^ conidia/mL [56]. All seedlings were placed in a growth chamber at 25 °C with a relative humidity of >90% and a 16 h/8 h light/dark photoperiod. Leaf samples were harvested before inoculation and on the 9th and 15th days after inoculation. Three biological replicates were included.

### 4.2. Microscopy Observations of C. fulvum in Ont7813 and MM

Based on a modified version of Faoro’s method [51], we used lactophenol trypan blue staining to observe the interaction process of *C. fulvum* with Ont7813 and MM. The leaves were sampled at 0, 9, 15, and 21 dai. A light microscope was used to further observe the changes associated with the HR, a scanning electron microscope (FEI, Florida, USA) was used to observe the surface morphology, and a transmission electron microscope (Hitachi Tokyo, Japan) was used to observe intracellular structural changes.

### 4.3. RNA Extraction and Illumina Sequencing

A total of 18 samples of Ont7813 and MM at 0, 9, and 15 dai were harvested for RNA-seq analysis. To simplify the description, the susceptible line MM at 0, 9, and 15 dai is referred to as MM-0, MM-9, and MM-15, and the resistant line Ont7813 at 0, 9, and 15 dai is referred to as Cf13-0, Cf13-9, and Cf13-15; for each treatment, three biological replicates were included.

The RNA libraries were sequenced on the Illumina sequencing platform by Genedenovo Biotechnology Co., Ltd. (Guangzhou, China); the following detailed experimental methods were provided by the company. Total RNA was extracted from the leaf samples, rRNA was removed using a conventional kit, and the mRNA was enriched. We further reverse transcribed the enriched mRNA to form double-stranded cDNA. After repairing the double ends of the cDNA, we performed ligations and PCR amplification to construct libraries. The libraries were then sequenced using an Illumina HiSeq 2500 machine (Illumina, CA, USA), and 150-bp paired-end reads were generated.

### 4.4. Mapping Reads and DEGs

FastQ (https://github.com/OpenGene/fastp, accessed on 15 January 2020) was used to filter the raw reads. The raw reads were cleaned by removing reads with adapters and all reads with poly-A tails. Low-quality reads (reads with more than 50% of bases having a Q value ≤ 20 or an ambiguous sequence content (“N”) that exceeded 10%) were also removed.

The clean reads were mapped to the *S. lycopersicum* reference genome sequence (the tomato genome version SL 4.0 and annotation ITAG 4.0). The fragments per kilobase of transcript per million mapped reads (FPKM) method was used to calculate gene expression. Bowtie2 [57] was used to compare the sequences of the clean reads to the sequence information in a ribosomal database. The ribosomal reads that were mismatched in the alignment were removed, and the remaining unmapped reads were used for subsequent transcriptome analysis. HISAT was used to align the paired-end clean reads to the reference genome [58]. DEGs were detected using DESeq2 [48] based on the negative binomial distribution model and BH. Genes with an adjusted FDR of <0.05 and a |log2FC| > 1 were defined as significantly differentially expressed. Novel transcripts were reconstructed using StringTie [59].

### 4.5. GO Enrichment and KEGG Pathway Analysis of DEGs

GO [49] enrichment analysis provides all the GO terms that are significantly enriched in DEGs compared with the genome background and filters the DEGs that correspond to biological functions. KEGG [60] is a major public pathway-related database [61]. GO terms and KEGG pathways with an adjusted |NES| > 1 and NOM *p* value of <0.05 were regarded as significantly enriched.

### 4.6. qRT–PCR Analysis

A total of 14 DEGs were selected to verify the expression profiles obtained by RNA-seq. qRT–PCR was performed in accordance with AceQ^®^ qPCR SYBR^®^ Green Master Mix (Vazyme, Nanjing, China), and the reactions were carried out on a qTOWER3G Detection System (Analytik Jena AG, Jena, Germany). Primer 5.0 software was used to design the primers (Table S2). The expression data were analyzed using the 2^−^^△△CT^ method [62], and the *EFa1* gene was used for normalization [63].

### 4.7. Measurements of JA and SA Hormone Contents

After inoculation, the two tomato cultivars were sampled at 0, 9, and 15 dai. Endogenous SA and JA were extracted from leaves according to the modified methods of Llugany et al. (2013) [64]. The SA and JA contents were measured via liquid chromatography–mass spectrometry (LC–MS) according to the manufacturer’s instructions (SCIEX, Redwood City, CA, USA).

## 5. Conclusions

In this study, a total of 696 novel genes were identified after StringTie was used to reconstruct transcripts that were not annotated in the reference genome (or for a set of reference genes). The DEG analysis showed a large difference between resistant and susceptible plants at the early response stage, which is very important for plant immunity. Moreover, early Ca^2+^ signaling processes differ between compatible and incompatible interactions of *C. fulvum* infection in Ont7813 and MM plants. The “plant hormone signal transduction” pathway, the “plant–pathogen interaction” pathway, and the “MAPK signaling pathway-plant” pathway contain many key regulatory components of the *Cf-13*-mediated resistance response processes. Particularly during the early stage, both PTI and ETI participate in the *Cf-13*-mediated resistance response. Ultimately, the hormone response balance between JA and SA may play a key role in the underlying regulatory activity of *Cf-13* against *C. fulvum* infection.

## Figures and Tables

**Table 1:** Summary of sequencing reads after filtering.

Sample	Raw Data (Mb)	Clean Data (Mb) (%)	Clean Bases (Gb)	AF_Q20 (%)	AF_Q30 (%)	AF_GC (%)
MM-0-1	22.75	22.73 (99.91%)	3.39	97.48%	92.75	44.24
MM-0-2	25.00	24.97 (99.91%)	3.73	98.41%	94.95	44.57
MM-0-3	28.50	28.47 (99.91%)	4.24	98.24%	94.61	44.59
MM-9-1	22.91	22.89 (99.89%)	3.41	97.97%	93.87	44.36
MM-9-2	30.66	30.63 (99.91%)	4.58	98.17%	94.24	44.73
MM-9-3	23.64	23.61 (99.88%)	3.52	97.86%	93.62	45.00
MM-15-1	25.19	25.17 (99.89%)	3.76	97.98%	93.95	44.76
MM-15-2	25.05	25.02 (99.88%)	3.72	98.07%	94.20	45.00
MM-15-3	26.87	26.84 (99.89%)	4.01	98.23%	94.50	44.92
Cf13-0-1	29.72	29.69 (99.91%)	4.43	98.28%	94.72	44.15
Cf13-0-2	24.60	24.58 (99.90%)	3.66	97.95%	93.80	44.62
Cf13-0-3	25.37	25.35 (99.90%)	3.79	97.93%	93.86	44.46
Cf13-9-1	22.99	22.96 (99.87%)	3.42	97.91%	93.77	44.33
Cf13-9-2	29.90	29.86 (99.88%)	4.46	97.93%	93.82	44.15
Cf13-9-3	23.09	23.06 (99.89%)	3.44	98.08%	94.18	44.24
Cf13-15-1	27.03	27.00 (99.90%)	4.02	98.12%	94.33	44.31
Cf13-15-2	31.16	31.13 (99.91%)	4.64	98.21%	94.50	44.19
Cf13-15-3	23.12	23.10 (99.92%)	3.44	98.28%	94.71	45.26

## Data Availability

The raw sequencing data of this article are stored in the NCBI Sequence Read Archive under accession numbers SRR18426570 to SRR18426587.

## Supplementary Materials

The following supporting information can be downloaded at: https://www.mdpi.com/article/10.3390/ijms23094844/s1.
