# Supplementary material for: Transcriptome Analysis of the Cf-13-Mediated Hypersensitive Response of Tomato to Cladosporium fulvum Infection
Source: Int J Mol Sci. 2022 Apr 27;23(9):4844. doi: 10.3390/ijms23094844 (PMC9102077; doi:10.3390/ijms23094844)
Supplement: Supplementary file 1 [file ijms-23-04844-s001.zip › ijms-1672821-supplementary.pdf]

Table S1 Top 20 KEGG pathways

ID	pathway name	class	Pvalue	per	ratio	fg_num	bg_num
ko01110	Biosynthesis of secondary metabolites	Metabolism	1.41E-05	35.071	0.046	74	1596
ko00941	Flavonoid biosynthesis	Metabolism	0.000189	4.739	0.118	10	85
ko00260	Glycine, serine and threonine metabolism	Metabolism	0.000377	4.265	0.118	9	76
ko04016	MAPK signaling pathway - plant	Environmental Information Processing	0.000833	8.531	0.068	18	265
ko00073	Cutin, suberine and wax biosynthesis	Metabolism	0.001041	2.844	0.15	6	40
ko04626	Plant-pathogen interaction	Organismal Systems	0.001244	11.848	0.057	25	441
ko00945	Stilbenoid, diarylheptanoid and gingerol biosynthesis	Metabolism	0.001593	3.791	0.107	8	75
ko00900	Terpenoid backbone biosynthesis	Metabolism	0.007063	2.844	0.103	6	58
ko00670	One carbon pool by folate	Metabolism	0.0175	1.422	0.158	3	19
ko00360	Phenylalanine metabolism	Metabolism	0.017751	3.791	0.071	8	112
ko01100	Metabolic pathways	Metabolism	0.022115	50.237	0.034	106	3078
ko00909	Sesquiterpenoid and triterpenoid biosynthesis	Metabolism	0.027402	1.896	0.103	4	39
ko04075	Plant hormone signal transduction	Environmental Information Processing	0.033685	9.005	0.046	19	409
ko01040	Biosynthesis of unsaturated fatty acids	Metabolism	0.037547	1.896	0.093	4	43
ko00940	Phenylpropanoid biosynthesis	Metabolism	0.046441	6.635	0.049	14	288
ko00564	Glycerophospholipid metabolism	Metabolism	0.050028	3.318	0.062	7	113
ko00561	Glycerolipid metabolism	Metabolism	0.052474	2.844	0.066	6	91
ko00591	Linoleic acid metabolism	Metabolism	0.053233	1.422	0.103	3	29
ko00430	Taurine and hypotaurine metabolism	Metabolism	0.054979	0.948	0.154	2	13
ko00760	Nicotinate and nicotinamide metabolism	Metabolism	0.057902	1.422	0.1	3	30

Table S2 Primers used for qRT-PCR analysis.

Primer name	Forward primer sequence (5'-3')	Reverse primer sequence (5'-3')
Solyc03g098210.4	GAACAGACTCTTCCAGCCCC	CTCAGTCTCTGTTTTGATTGGCA
Solyc06g051920.4	TCTAAAAGCTGTGGCTGATGAT	CCTGTCTGGCACTCTTGCTT
Solyc02g092930.1	TGGCCCGAGAAATTGGTCTC	AGTTGATCGGCGAGTTCGTT
Solyc03g113390.3	AGGATGGGTCTCTCCAGCTT	CCACTCAAGCTCATTTTCCCCT
Solyc02g072470.4	TGCTGTCACATGATTACAGGGG	AGCTGGTTTCTCTCCATGCC
Solyc03g006080.4	GCATGCTTCCAGTATCTGCG	TAAGTTGCCAATTGACGCGG
Solyc03g006100.4	CACGCTGAGCATGTTATCGG	GAAACAGTGACATTTATTGCAACC
Solyc10g011910.4	GCCGGCGACATCAGTTTTAC	TAAGCTGTTGCGACGGTGAT
Solyc06g068680.3	GATATCCGCGATGACACCGT	CTGCGCTTTTTCTGCAGGTT
Solyc02g094000.1	ATGAGGAAGCAAAGGCTGCT	TGAACGCCTCCTTCAACTCG
Solyc04g018110.1	AGATCCCTTTCCCGATCCGA	ATGTCGGACCACTCATCAGC
Solyc10g079420.1	GTCCGTAACCACTCCGACAA	ATCTCGGAATCACACGACGG
Solyc11g071740.2	ATGGGGACGGATTATTGGGC	ATGCTCCCATCAACTCACCC
Solyc02g088090.1	CTTGCTAATTCATGGGGCGG	CACACACTTGGGTTTCAGCA
EFα1	CCACCAATCTTGTACACATCC	AGACCACCAAGTACTACTGCAC

Table S3 DEGs in the significantly enriched KEGG pathway “Plant-pathogen interaction”

Gene ID	Symbol	log2 Fold-change					
		**MM 0-vs-9 dai**	**MM 9-vs-15 dai**	**MM 0-vs-15 dai**	**Cf-13 0-vs-9 dai**	**Cf-13 9-vs-15 dai**	**Cf-13 0-vs-15 dai**
MSTRG.6105	At3g47570	-0.28	-0.17	-0.45	1.91	-1.85	0.05
MSTRG.9104	XA21	1.37	-0.72	0.65	3.45	-2.24	1.21
Solyc02g072440.4	XA21	2.12	-0.46	1.65	4.30	1.78	6.08
Solyc02g079280.3	MYB102	0.93	-1.52	-0.59	1.91	-2.15	-0.25
Solyc02g081040.4	LYK3	0.06	-0.42	-0.35	1.72	-1.38	0.34
Solyc02g083850.3	CPK28	0.83	-1.26	-0.43	1.97	-1.70	0.27
Solyc02g092930.1	MYB73	1.16	-0.63	0.53	-3.09	0.61	-2.48
Solyc03g006080.4	At3g47570	0.41	-0.72	-0.31	2.78	-2.83	-0.05
Solyc04g014645.1	XA21	1.32	-1.60	-0.28	5.59	-4.22	1.37
Solyc04g014883.1	XA21	0.90	-1.48	-0.58	5.08	-3.44	1.64
Solyc05g050360.3	CNGC3	0.97	-1.55	-0.57	1.97	-1.61	0.36
Solyc06g005330.3	MYB48	-0.72	0.06	-0.66	-2.26	2.34	0.08
Solyc06g048735.1	BAM1	2.62	-3.52	-0.90	3.94	-4.08	-0.14
Solyc06g048740.3	XA21	3.19	-2.13	1.07	4.91	-3.69	1.22
Solyc06g068960.1	CML27	0.90	-0.75	0.15	1.96	-1.43	0.53
Solyc06g073830.1	--	1.68	-0.99	0.69	-3.25	0.61	-2.64
Solyc06g083390.4	RIN4	0.32	-0.75	-0.43	1.72	-2.05	-0.33
Solyc07g049180.3	LYK3	0.06	-0.76	-0.70	1.82	-1.38	0.45
Solyc08g069140.4	CNGC14	0.99	-0.70	0.30	1.34	-0.13	1.21
Solyc09g006005.1	PR1B1	1.34	-0.60	0.74	1.00	0.63	1.63
Solyc09g007010.1	PR1B1	0.29	-3.03	-2.74	-5.82	2.33	-3.49
Solyc10g011910.4	WRKY22	2.25	-1.49	0.76	-3.61	-0.76	-4.38
Solyc11g018610.2	CPK4	0.59	-0.42	0.18	1.91	-1.44	0.47
Solyc11g069580.2	CNGC15C	0.12	-0.24	-0.12	2.07	-1.34	0.73
Solyc11g071740.2	CML38	2.36	-1.62	0.75	-4.99	-0.04	-5.02

Table S4 DEGs in the significantly enriched KEGG pathway “MAPK signaling pathway–plant”

Gene ID	Symbol	log2 Fold-change					
		**MM 0-vs-9 dai**	**MM 9-vs-15 dai**	**MM 0-vs-15 dai**	**Cf-13 0-vs-9 dai**	**Cf-13 9-vs-15 dai**	**Cf-13 0-vs-15 dai**
MSTRG.6105	At3g47570	-0.28	-0.17	-0.45	1.91	-1.85	0.05
MSTRG.9104	XA21	1.37	-0.72	0.65	3.45	-2.24	1.21
Solyc01g095080.3	ACS2	-2.60	-1.14	-3.74	-5.64	1.73	-3.91
Solyc02g072440.4	XA21	2.12	-0.46	1.65	4.30	1.78	6.08
Solyc03g006080.4	At3g47570	0.41	-0.72	-0.31	2.78	-2.83	-0.05
Solyc03g007050.3	ERL1	1.35	-0.21	1.14	1.95	0.65	2.60
Solyc04g007710.3	MPK9	0.95	-0.85	0.10	1.02	0.24	1.25
Solyc04g014645.1	XA21	1.32	-1.60	-0.28	5.59	-4.22	1.37
Solyc04g014883.1	XA21	0.90	-1.48	-0.58	5.08	-3.44	1.64
Solyc06g048735.1	BAM1	2.62	-3.52	-0.90	3.94	-4.08	-0.14
Solyc06g048740.3	XA21	3.19	-2.13	1.07	4.91	-3.69	1.22
Solyc06g051940.4	PP2C51	3.54	-0.78	2.76	3.51	-2.05	1.46
Solyc09g006005.1	PR1B1	1.34	-0.60	0.74	1.00	0.63	1.63
Solyc09g007010.1	PR1B1	0.29	-3.03	-2.74	-5.82	2.33	-3.49
Solyc10g011910.4	WRKY22	2.25	-1.49	0.76	-3.61	-0.76	-4.38
Solyc10g055810.2	CHI9	1.27	-1.67	-0.40	-2.05	2.15	0.10
Solyc11g005720.1	MKS1	1.39	-0.84	0.55	-1.73	-0.62	-2.36
Solyc12g009020.2	MKK2	0.38	-0.81	-0.43	1.47	-1.15	0.33

Table S5 DEGs in the significantly enriched KEGG pathway “Plant hormone signal transduction”

**Ggne ID**	**Symbol**	**log2 Fold-change**					
		**MM 0-vs-9 dai**	**MM 9-vs-15 dai**	**MM 0-vs-15 dai**	**Cf-13 0-vs-9 dai**	**Cf-13 9-vs-15 dai**	**Cf-13 0-vs-15 dai**
Solyc01g098400.3	AHP1	0.26	-2.33	-2.07	-0.99	-0.26	-1.25
Solyc03g082510.1	SAUR32	0.51	-2.21	-1.71	-0.23	-0.55	-0.77
Solyc03g082520.1	SAUR32	1.82	-3.87	-2.05	1.58	-0.91	0.67
Solyc03g082530.1	SAUR32	3.81	-2.53	1.29	2.79	-1.39	1.40
Solyc04g078840.3	ABF2	-0.22	1.46	1.25	0.77	-0.99	-0.22
Solyc05g009660.4	PAN	-0.35	1.42	1.07	-0.54	-0.41	-0.94
Solyc06g008780.3	TIR1	0.07	0.76	0.82	-0.84	0.00	-0.84
Solyc06g051940.4	PP2C51	-1.71	3.51	1.80	3.79	-0.78	3.01
Solyc06g053290.1	SAUR32	0.12	1.96	2.08	0.26	-1.34	-1.09
Solyc07g044980.3	NPR3	0.05	1.25	1.30	0.28	-0.77	-0.49
Solyc08g007230.3	ERF1A	2.78	-4.34	-1.57	-2.32	3.92	1.60
Solyc09g006005.1	PR1B1	0.62	1.00	1.62	-0.90	-0.60	-1.51
Solyc09g007010.1	PR1B1	2.53	-5.82	-3.29	1.26	-3.03	-1.78
Solyc09g009490.4	ABI5	0.22	2.51	2.73	-1.71	-0.46	-2.16
Solyc09g065850.4	AUX22D	2.39	-2.11	0.28	0.84	-0.91	-0.07
Solyc11g011030.2	--	-2.06	2.89	0.83	1.30	-0.51	0.78
Solyc11g069093.1	SAUR50	-0.65	4.77	4.12	0.58	0.12	0.70
Solyc12g005310.2	GH3.6	0.38	-1.81	-1.43	0.52	-0.63	-0.11
Solyc12g049400.2	TIFY10B	-0.85	1.82	0.97	1.53	-0.29	1.24

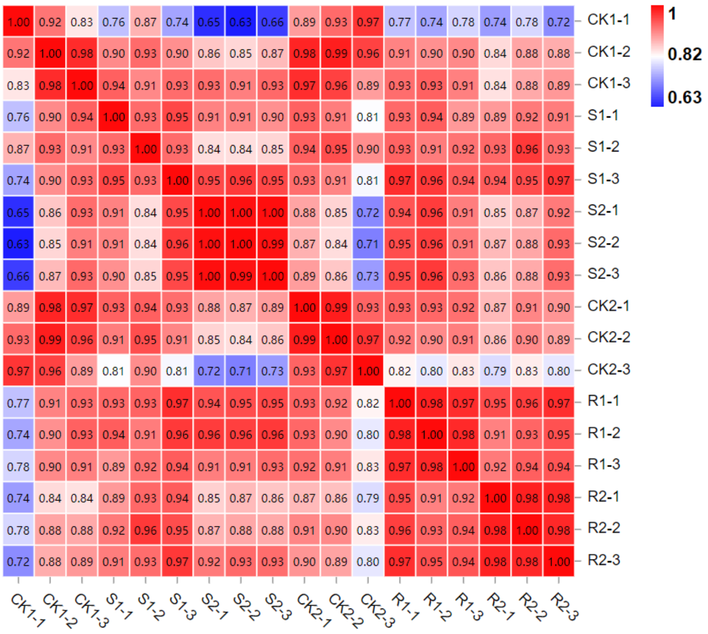

Figure S1. Pearson correlation coefficients of the 18 samples. The correlation coefficients between two samples are visualized as heat maps.

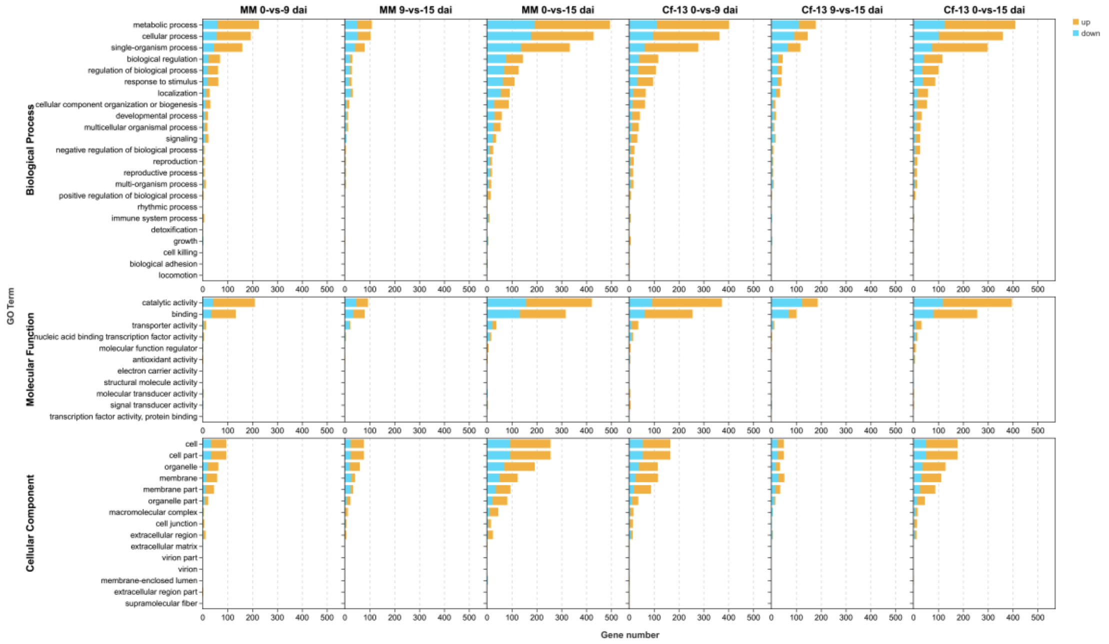

Figure S2. GO classification of DEGs.

The X axis represents the number of DEGs. The Y axis represents the GO terms. 0–9 dai: comparison between 0 and 9 dai; 9–15 dai: comparison between 9 and 15 dai; 0–15 dai: comparison between 0 and 15 dai; dai: days after inoculation.

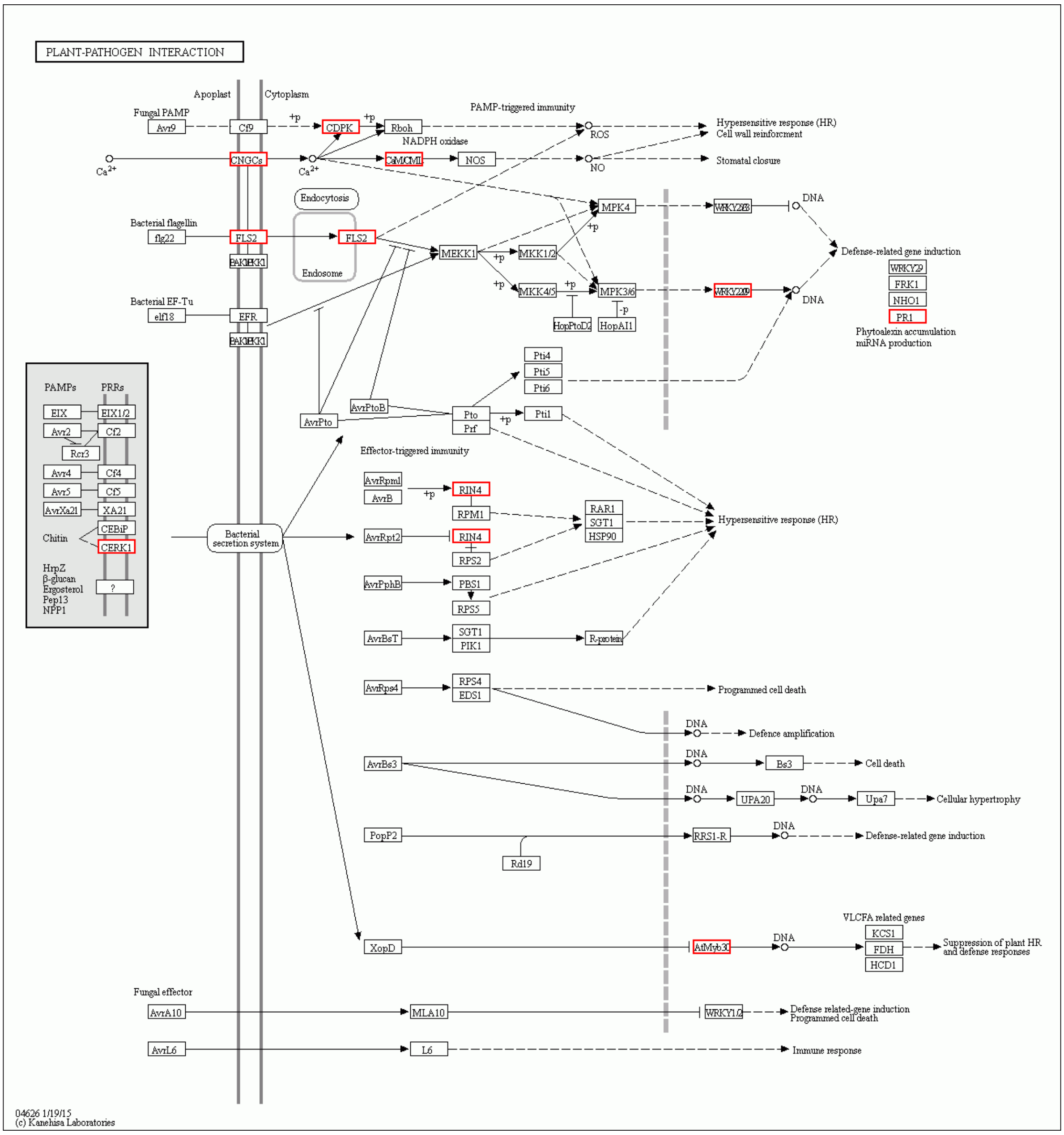

Figure S3. map04626, plant-pathogen interaction

DEGs in the significantly enriched KEGG pathway “plant-pathogen interaction” are mapped in Figure S3

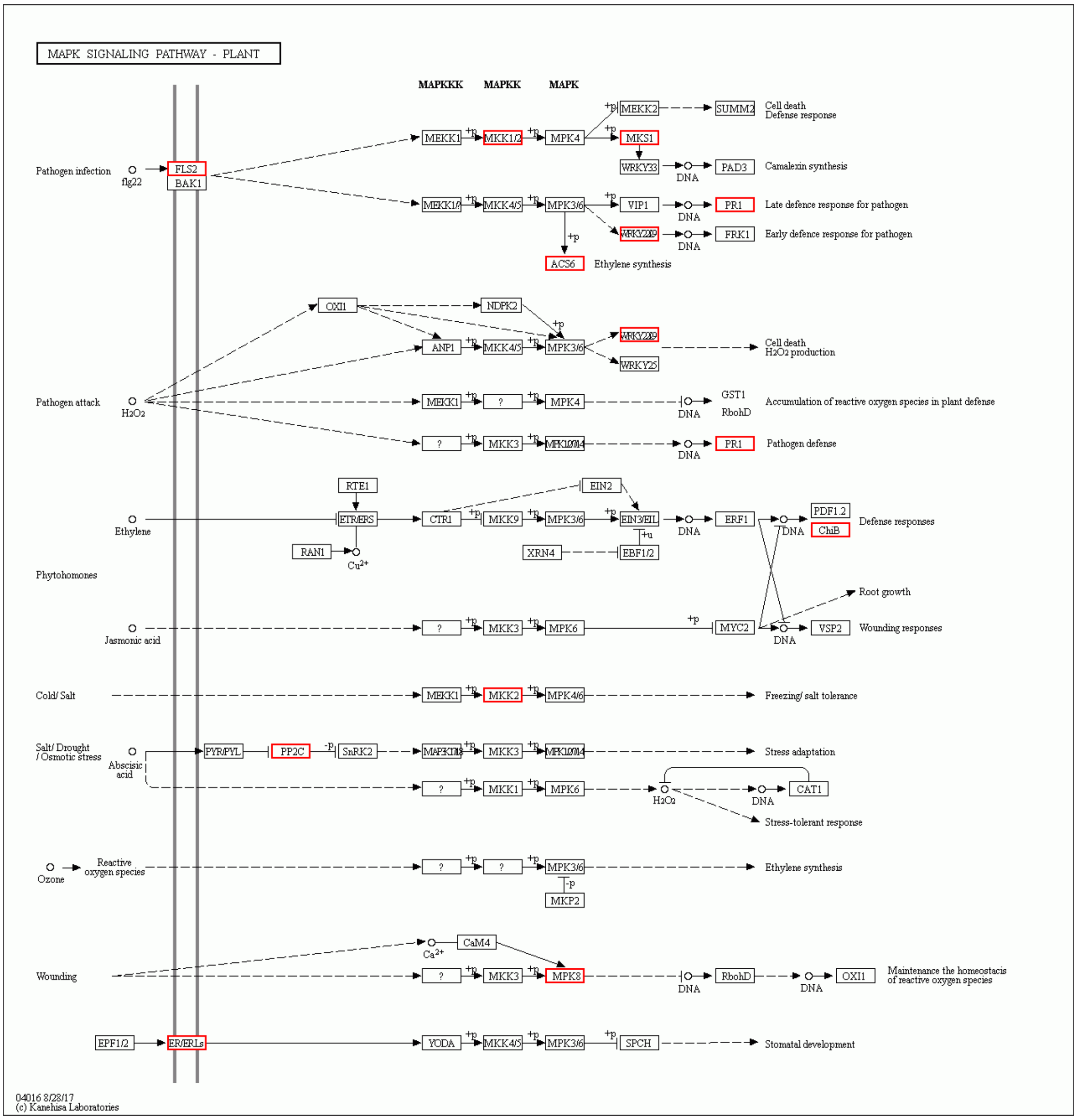

Figure S4. map04016, MAPK signaling pathway–plant

DEGs in the significantly enriched KEGG pathway “MAPK signaling pathway–plant” are mapped in Figure S4

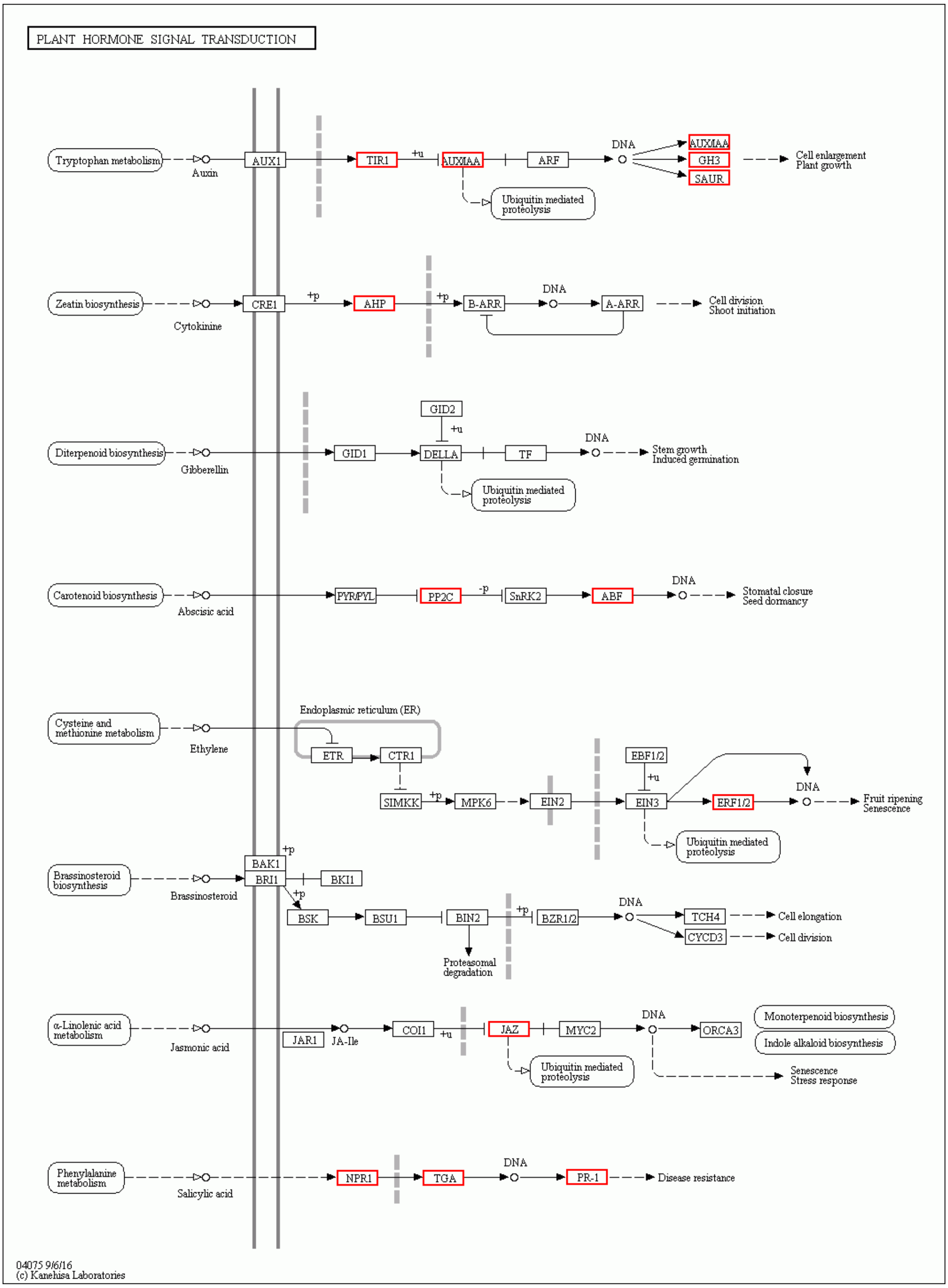

Figure S5. map04075, plant hormone signal transduction

DEGs in the significantly enriched KEGG pathway “plant hormone signal transduction” are mapped in Figure S5
